# Potential antioxidative and anti-hyperuricemic components in *Rodgersia podophylla* A. Gray revealed by bio-affinity ultrafiltration with SOD and XOD

**DOI:** 10.3389/fphar.2023.1298049

**Published:** 2023-11-08

**Authors:** Can Liang, Yongbing Xu, Minxia Fan, Felix Wambua Muema, Guilin Chen, Mingquan Guo, Guangwan Hu

**Affiliations:** ^1^ Key Laboratory of Plant Germplasm Enhancement and Specialty Agriculture, Wuhan Botanical Garden, Chinese Academy of Sciences, Wuhan, China; ^2^ University of Chinese Academy of Sciences, Beijing, China; ^3^ Sino-Africa Joint Research Center, Chinese Academy of Sciences, Wuhan, China; ^4^ Hubei Jiangxia Laboratory, Wuhan, China

**Keywords:** *Rodgersia podophylla*, UF-LC-MS, antioxidative, anti-hyperuricemic, superoxide dismutase, xanthine oxidase

## Abstract

*Rodgersia podophylla* A. Gray (*R. podophylla*) is a traditional Chinese medicine with various pharmacological effects. However, its antioxidant and anti-hyperuricemia components and mechanisms of action have not been explored yet. In this study, we first assessed the antioxidant potential of *R. podophylla* with 2,2-diphenyl-1-picrylhydrazyl (DPPH), 2,2′-azino-bis(3-ethylbenzothiazoline-6-sulfonic acid) (ABTS) and ferric ion reducing antioxidant power (FRAP) assays. The results suggested that the ethyl acetate (EA) fraction of *R. podophylla* not only exhibited the strongest DPPH, ABTS radical scavenging and ferric-reducing activities, but also possessed the highest total phenolic and total flavonoid contents among the five fractions. After that, the potential superoxide dismutase (SOD) and xanthine oxidase (XOD) ligands from the EA fraction were quickly screened and identified through the bio-affinity ultrafiltration liquid chromatography-mass spectrometry (UF-LC-MS). Accordingly, norbergenin, catechin, procyanidin B2, 4-O-galloylbergenin, 11-O-galloylbergenin, and gallic acid were considered to be potential SOD ligands, while gallic acid, 11-O-galloylbergenin, catechin, bergenin, and procyanidin B2 were recognized as potential XOD ligands, respectively. Moreover, these six ligands effectively interacted with SOD in molecular docking simulation, with binding energies (BEs) ranging from −6.85 to −4.67 kcal/mol, and the inhibition constants (Ki) from 9.51 to 379.44 μM, which were better than the positive controls. Particularly, catechin exhibited a robust binding affinity towards XOD, with a BE value of −8.54 kcal/mol and Ki value of 0.55 μM, which surpassed the positive controls. In conclusion, our study revealed that *R. podophylla* possessed remarkable antioxidant and anti-hyperuricemia activities and that the UF-LC-MS method is suitable for screening potential ligands for SOD and XOD from medicinal plants.

## 1 Introduction

*Rodgersia podophylla* A. Gray (*R. podophylla*), a member of the Saxifragaceae family, is primarily found in the Hubei, Sichuan, Yunnan, and Tibet of China. It grows in shady and wet places such as undergrowth, shrubland, meadows, and rock crevices, at altitudes range of 1,100–3,400 m (Zhang et al., 2005). The rhizome of *R. podophylla* is used as a traditional Chinese medicine and is known by several trade names, including Suogudan and Yantuo (Zhang et al., 2015). It has been used to treat enteritis, dysentery, dysmenorrhea, menorrhagia, rheumatoid arthritis, bruises, traumatic bleeding, and scrotal eczema (Yan et al., 2017). Modern pharmacological studies also indicated that *R. podophylla* exhibited antioxidant, antibacterial, immune-enhancing, hepatoprotective, antimalarial, anticancer, and anti-inflammatory properties (Xie et al., 2022). Researchers have recently conducted extensive studies on its chemical components and revealed to contain various chemical classes, such as phenylpropanoids, flavonoids, terpenes and their derivatives, steroids, and organic acids and their derivatives (Zhang et al., 2005; Xie et al., 2022).

Reactive oxygen species (ROS) are cellular metabolites, and are normally classified into two categories: free radicals, such as superoxide anion radicals (O_2_^•−^), and non-free radical species, such as hydrogen peroxide (Tomczyk and Malgorzata, 2021). ROS plays a crucial role in many important life processes in the human body, including cell growth (Brillo et al., 2021), proliferation, differentiation (Cheung and Vousden, 2022; Oka et al., 2022), energy supply (Yang and Lian, 2020), health, and aging (Alexey et al., 2020). However, excessive ROS in the body might expose cells to oxidative stress to inflict damage upon multiple organs, leading to a variety of health issues (Payne et al., 2013). To prevent organ damage from excess ROS, cells develop a protective system associated with redox enzymes, such as superoxide dismutase (SOD) and xanthine oxidase (XOD). Thereinto, SOD catalyzes the conversion of O_2_^•−^ to H_2_O_2_ and oxygen (O_2_) in the presence of transition metal ions, while H_2_O_2_ can be further decomposed into hydroxyl radicals (·OH) and hydroxide ions (OH^−^) (Zhuang et al., 2021). XOD is a flavoproteinase that catalyzes the production of uric acid and superoxide anion (O_2_^•−^) from xanthines and hypoxanthines (Chung et al., 1997). Excess uric acid and O_2_^•−^ produced by XOD metabolism are closely associated with hyperuricemia (Liu et al., 2021), gout (Wang et al., 2020), hepatitis (Chung and Yu, 2000), cancer (Chung et al., 1997; Mi et al., 2020), aging, cardiovascular disease (Yu and Cheng, 2020), and chronic obstructive pulmonary disease (Rumora et al., 2020). Therefore, SOD and XOD are the potential targets for the treatment of oxidative stress injury-related diseases in humans.

At present, *R. podophylla* has been reported to exert arrestive antioxidative (Kim et al., 2020; Pyo et al., 2020) and anti-hyperuricemic activities (Wang et al., 2012), but the specific components responsible for these effects have not been revealed. Bio-affinity ultrafiltration liquid chromatography-mass spectrometry (UF-LC-MS) has been widely used to screen and identify potential ligands for biotarget enzymes from complex samples (Chen et al., 2018; Fan et al., 2022). Therefore, it is feasible to use SOD and XOD as target enzymes to screen potential bioactive compounds in *R. podophylla* that may be effective against diseases caused by oxidative damage and hyperuricemia.

In this case, due to the complex and diverse chemical compositions of *R. podophylla*, it is necessary to first screen and select its active fraction. To accomplish this, the antioxidative capacities of different solvent extracts of *R. podophylla* were evaluated using three assays: DPPH (2,2-diphenyl-1-picrylhydrazyl), ABTS (2,2′-azinobis-(3-ethylbenzthiazoline-6-sulfonic acid)), and FRAP (ferric-reducing antioxidant power). The extracts included crude extract (CE), n-hexane (n-Hex), dichloromethane (DCM), ethyl acetate (EA), and water (H_2_O) fractions. At the same time, the total phenolic (TPC) and total flavonoid content (TFC) in different fractions of *R. podophylla* were detected to reveal their correlations with antioxidant activity. Then, the potential SOD and XOD ligands within the EA fraction were fast fished out with the UF-LC-MS method. Finally, molecular docking, along with the XOD inhibition assays *in vitro*, were employed to explain the interactions between the active components and the target enzymes. Consequently, this work provided a valuable reference for the development and utilization of *R. podophylla* as a natural antioxidant and anti-hyperuricemia drug or health supplement.

## 2 Materials and methods

### 2.1 Plant materials preparation and extraction

The rhizome of *R. podophylla* was collected in Xintang Township, Enshi City, Hubei Province, China, and was identified by Guangwan Hu, a senior taxonomist from the Key Laboratory of Plant Germplasm Enhancement and Specialty Agriculture (Wuhan Botanical Garden), Chinese Academy of Sciences. The voucher specimen (No. 20220705) was preserved in the herbarium of the Key Laboratory of Plant Germplasm Enhancement and Specialty Agriculture.

The dry, crushed rhizome (20 g) was soaked overnight in 200 mL of 50% ethanol at room temperature, then extracted using ultrasound for 50 min. This process was repeated three times, and the filtrate was collected. The filtrate was then concentrated using a rotary evaporator, and dried using a vacuum freeze dryer to obtain the crude extract of *R. podophylla* (CE, 4.32 g). 2 g of CE was dissolved in 100 mL of water and extracted consecutively with n-hexane (n-Hex, 100 × 3 mL), dichloromethane (DCM, 100 × 3 mL), and ethyl acetate (EA, 100 × 3 mL) to obtain n-Hex (24.3 mg), DCM (15.10 mg), EA (398.40 mg), and water (H_2_O, 75.15 mg) fractions, respectively. The obtained samples were stored in a sealed container and kept in a refrigerator at 4 °C for future use.

### 2.2 Chemicals and reagents

The chemical standards of gallic acid, bergenin, procyanidin B2 and catechin (Purity ≥98.0%) were bought from Chengdu Alfa Biotechnology Co., Ltd (Chengdu, China). Rutin was purchased from J&K Scientific Ltd. (Beijing, China), Folin-Ciocalteu reagent, 1,3,5-tri (2-pyridyl)-2,4,6-triazine (TPTZ), 2,2′-azinobis-(3-ethylbenzthiazoline-6-sulfonic acid) (ABTS), and ascorbic acid (vitamin C, Vc) were purchased from Sigma-Aldrich Corp (Shanghai, China). The acetonitrile (ACN) and methanol of HPLC grade were supplied by TEDIA Company Inc (Fairfield, OH, United States). All other analytical solvents and chemicals were purchased from Sinopharm Chemical Reagent Co., Ltd. (Shanghai, China). Superoxide dismutase (SOD) and xanthine oxidase (XOD) were bought from Shanghai Yuanye Bio-Technology Co., Ltd. (Shanghai, China). Ultrafiltration membranes (0.5 mL, 30 kDa) were purchased from Millipore Co. Ltd. (Bedford, MA, United States). Water (ultrapure grade) for HPLC and HPLC-UV-ESI-MS/MS analyses was prepared with EPED (Nanjing EPED Technology Development Co., Ltd. Nanjing, China).

### 2.3 Instruments

HPLC-UV/ESI-MS/MS was conducted with a Thermo Accela 600 series HPLC system coupled with a TSQ Quantum Access MAX mass spectrometer (Thermo Fisher Scientific, San Jose, CA, United States). As for the HPLC analysis, an Agilent 1220 LC (Santa Clara, CA, United States) with a RP-C18 column (Waters Symmetry RP-C18, 4.6 mm × 250 mm, 5 μm) was applied, and the UV absorbance was recorded by UV/VIS Spectrophotometer (UV1100, Shanghai, China). Centrifugation of samples was carried out by low-temperature high-speed centrifuge (Centrifuge 5810R, Eppendorf, Germany).

### 2.4 Determinations of antioxidant activity of *R. podophylla*

#### 2.4.1 DPPH free radical scavenging activity

The DPPH radical scavenging activity of *R. podophylla* samples was determined based on a previously reported study by Muema et al. (Muema et al., 2022). Briefly, 10 μL of sample or positive control solution (Vc, 46.875–3,000 μM) was mixed with 190 μL DPPH (100 μM) in a 96-well plate. The mixture was then incubated for 30 min in the dark at room temperature. The absorbance at 517 nm was measured with a multifunctional microplate reader. Methanol was used as a blank control, and all samples and control were tested in triplicate (n = 3). The DPPH radical scavenging activity was calculated using the formula:

$$scavenging\;effect\;\left(\%\right)=\frac{A_{c}-A_{s}}{A_{c}}\times 100\%$$

where A_c_ and A_s_ represent the absorbance values of the blank control and the tested sample or positive control, respectively. The IC_50_ value represents the concentration of the tested sample or positive control when the inhibition rate of DPPH radicals is 50%.

#### 2.4.2 ABTS free radical scavenging activity

The ABTS free radical scavenging activity of different *R. podophylla* extracts was detected using a modified method described by Fan et al. (Fan et al., 2022). An ABTS solution (7 mM aqueous solution) was mixed with potassium persulfate (4.9 mM aqueous solution) in equal volumes (v/v) and allowed to react in the dark for 12–16 h to create a working solution (ABTS^+^). The ABTS^+^ solution was then diluted with methanol to ensure that its absorbance at 734 nm was approximately 0.700 ± 0.100. Next, 190 μL of the ABTS^+^ solution was mixed with 10 μL of the sample and the absorbance was measured at 734 nm after incubating in the dark for 30 min. Methanol and Vc (31.25–1,000 μM) were used as blank and positive controls, respectively. The calculation of ABTS scavenging activity results followed the previous format in DPPH part.

#### 2.4.3 Ferric-ion-reducing antioxidant power assay (FRAP)

The determination of ferric-iron-reducing antioxidant power (FRAP) was performed using a slightly modified method described by Xu et al. (Xu et al., 2019). The FRAP reagent (Fe^3+^-TPTZ solution) containing 20 mM FeCl_3_.6H_2_O, 10 mM TPTZ, and 300 mM acetate buffer (3.1 g C_2_H_3_NaO_2_.3H_2_O and 16 mL C_2_H_4_O_2_, pH 3.6) was prepared in a ratio of 1:1:10 (v/v/v) and stored at 37°C. Next, 10 μL of appropriately diluted sample, 30 μL of ultrapure water, and 260 μL of fresh Fe^3+^-TPTZ solution were added to a 96-well plate and incubated at 37°C for 10 min. The absorbance of the mixture at 593 nm was then measured using the multifunctional microplate reader, with three replicates per sample. FeSO_4_.7H_2_O (62.5, 125, 250, 500, 1,000, and 2000 μM) was used as a standard to establish a calibration curve, and FRAP activity was expressed as the equivalent mM Fe^2+^ per Gram of sample (mM Fe^2+^/g sample).

### 2.5 Determination of phenolic constituents

#### 2.5.1 Determination of total phenolic content (TPC)

TPC was measured using the Folin-Ciocalteu method reported by Fan et al. (Fan et al., 2022). The reaction system was set up by sequentially adding 20 μL of the diluted sample, 20 μL of Folin-Ciocalteu reagent (diluted with pure water to 25%, v/v), 100 μL of sodium carbonate (Na_2_CO_3_, 1 M), and 20 μL of ultrapure water. The reaction was allowed to proceed for 1 h at room temperature in the dark. The absorbance was then recorded at 760 nm. Gallic acid solution (5–40 μg/mL) was used as a standard to establish a calibration curve. Results for TPC were expressed as milligrams of gallic acid equivalents per Gram of dry sample (mg GAE/g sample).

#### 2.5.2 Determination of total flavonoid content (TFC)

The TFC of *R. podophylla* ethanol extract and different fractions was determined using a slightly modified method described by Chen et al. (Chen et al., 2020a). A 96-well plate was prepared by adding 20 μL of sample solution and 10 μL of sodium nitrite solution (NaNO_2_, 5%, w/v) and incubated at room temperature for 6 min. Next, 12 μL of aluminum nitrate (Al(NO_3_)_3_, 10%, w/v) was added and incubated for an additional 6 min. Finally, 120 μL of sodium hydroxide solution (NaOH, 4%, w/v) and 58 μL of methanol solution were added, and the reaction was allowed to proceed for 15 min before measuring the absorbance at 510 nm. Rutin was used as the standard, and the results for TFC are expressed as milligrams of rutin equivalents per Gram of dry sample (mg RE/g sample).

### 2.6 Sample preparation and screening of the potential ligands of SOD and XOD with UF-LC-MS

Based on previous studies (Chen et al., 2020c; Fan et al., 2022), the UF-LC-MS method was applied to screen potential antioxidant components with high binding affinities to SOD and XOD in EA fractions. First, 10 mg of *R. podophylla* EA fraction was dissolved in 1 mL of Tris-HCl buffer solution (pH = 7.8) and sonicated for 30 min. Next, 180 μL of the sample solution was mixed with 20 μL of SOD (0.1 U/μL, pH 7.8) or XOD (0.2 U/μL, pH 6.8) and incubated at 37°C for 1 h. The mixed solutions were then transferred to a 30 kD ultrafiltration tube and centrifuged at 10,000 rpm for 10 min. The tube was washed three times with the appropriate PBS buffer to remove non-specifically bound components. Next, 200 μL of methanol (90%, v/v) was added and incubated for 20 min to release the enzyme-bound compounds, followed by centrifugation at 10,000 rpm for 10 min (n = 3). Finally, the ultrafiltrate was collected, dried using a termovap sample concentrator, and redissolved in 50 μL of 90% methanol for further analysis. As a negative control, an equal amount of enzyme was incubated in boiling water (100°C) for 10 min and treated in the same manner as the active enzyme group.

### 2.7 HPLC-UV/ESI-MS/MS analysis

The chemical compositions of the *R. podophylla* EA fraction were currently analyzed and identified with a Thermo Accela 600 HPLC system in combination with a TSQ Quantum Access MAX MS equipped with an ESI interface. Separation was performed using a Waters Sunfire RP-C18 column (4.6 mm × 250 mm, 5 μm; Waters, Wexford, Ireland). The mobile phases for HPLC elution were 0.1% (v/v) formic acid aqueous solution (A) and ACN (B). The elution conditions were as follows: 1%–5% (B) in 0–5 min, 5%–14% (B) in 5–25 min, and 14%–20% (B) in 25–60 min. The injection volume was 10 μL, the flow rate was 0.8 mL/min, and the wavelength was set at 310 nm (Wang et al., 2012). The ESI-MS/MS analysis was performed in the negative ion mode under the following conditions: the temperatures of vaporiser and capillary were 350 °C and 250 °C, respectively; the gas pressure of sheath gas (nitrogen gas, N_2_) and axu gas (helium, He) was 40 psi and 10 psi, respectively; the source voltage was 3000 V and the cone voltage and collision energy were 40 V and 10 V, respectively; mass range (m/z) was from 150 to 1,500, spray voltage was 3 kV. MS data (mass range from m/z 100–1,000) was obtained in the full-scan mode. The preliminary identification of compounds in the EA fraction of *R. podophylla* was carried out by comparing the parent ions, MS fragments, and retention times with the references.

### 2.8 Molecular docking study

The interactions between the ligands and target enzymes were further elucidated through molecular docking. This process was based on previous studies by Fan et al. (Fan et al., 2022; Rakotondrabe et al., 2022) with minor modifications, using software such as AutoDock Tools 1.5.6, PyMOL and the Discovery Studio 4.1 software. The structures of SOD (PDB 1CBJ) and XOD (PDB 1FIQ) were obtained from the RSCB Protein Database (www.rcsb.org), while the 3D structure of the ligand was downloaded from the Traditional Chinese Medicine Systems Pharmacology Database and Analysis Platform (old.tcmsp-e.com). Next, water molecules and ligand fractions were removed using the PyMOL. Hydrogen atoms were added to proteins and ligands using AutoDock Tools, which was also used to calculate charges and perform other processing of their 3D structures. The coordinates of the active sites of SOD and XOD were (X: 6.640; Y: 23.974; Z: 58.655) and (X: 28.671; Y: 29.977; Z: 101.417) (Fan et al., 2022), respectively. A grid box was centered on the active sites of the receptors, with dimensions of 60 Å × 60 Å × 60 Å. Molecular docking analysis between ligands and receptors was then performed using AutoDock Tools, with 50 independent runs of the genetic algorithm and other default parameters. The docking conformations were ranked according to their energy scores.

### 2.9 Validation of potential ligands activity by XOD inhibition experiment

The XOD inhibition experiment was performed using a modified method previously reported by Wang et al. (Wang et al., 2012). In a 96-well plate, 90 μL of phosphate buffer (0.1 M, pH = 7.4), 20 μL of XOD (0.5 U/mL), and 10 μL of sample solution or positive control allopurinol solution dissolved in phosphate buffer were added and incubated at 37 °C for 30 min. Then, 80 μL of substrate (xanthine solution dissolved in phosphate buffer, 0.4 mM) was added and the plate was incubated at 37°C in the dark for 15 min. Absorbance was measured at 295 nm. Each well had a blank control without enzyme and a background control for 100% enzyme activity (only the solvent with the enzyme and the substrate). All samples and controls were tested in triplicate (n = 3). XOD inhibition was calculated as follows:

$$Inhibition\;\left(\%\right)=\left(1-\frac{A_{Sample}-A_{Blank}}{A_{Background}\;–\;A_{Blank}}\right)\times 100\%$$

where A_Sample_, A_Blank_ and A_Background_ are defined as the absorbance of the test sample (with the enzyme), the blank (the test sample without the enzyme) and the 100% enzyme activity (only the solvent with the enzyme and the substrate), respectively. The extent of inhibition was expressed as the concentration of sample needed to inhibit XOD activity by 50% (IC_50_).

### 2.10 Statistical analysis

All data in this work were measured in triplicate and were expressed as mean ± standard deviation (SD). The IC_50_ values were calculated by plotting the percentages of scavenging activities or inhibition rates against the sample concentrations (six different concentration gradients in triplicate). Statistical analysis was performed using software of SPSS 25.0 (IBM Corp., New York, NY, United States), Origin 2021 (OriginLab Corporation, Northampton, MA, United States) and GraphPad Prism 9.0 (GraphPad Software Inc., San Diego, CA, United States).

## 3 Results and discussion

### 3.1 Antioxidant activities of *R. podophylla*

**FIGURE 1:**
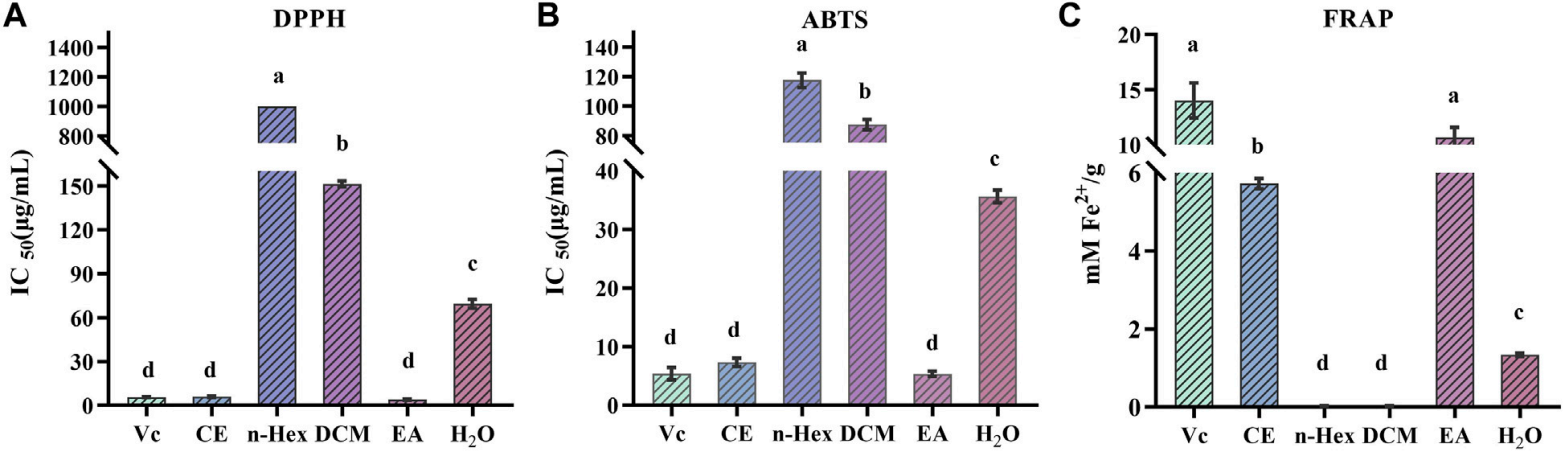
Antioxidant activity of n-hexane (n-Hex), dichloromethane (DCM), ethyl acetate (EA), H_2_O and crude extracts (CE) of *R. podophylla*. **(A)** the IC_50_ value of DPPH radical scavenging assay, **(B)** the IC_50_ value of ABTS radical scavenging assay, **(C)** the FRAP assay. Mean values with different letters **(A–C)** were significantly different at a level of *p* < 0.05 (n = 3) by DMRT (Duncan’s multiple range test).

Due to the multiple ROS scavenging patterns and the complexity of natural phytochemicals, three representative experiments, including DPPH, ABTS, and FRAP, were selected to evaluate and compare the antioxidant activity of different *R. podophylla* extracts (Fan et al., 2020). As shown in Figure 1, the EA fraction showed the strongest scavenging effect on DPPH and ABTS free radicals compared to the other four fractions, with IC_50_ values of 3.863 ± 0.34 μg/mL and 5.537 ± 0.38 μg/mL, respectively. The IC_50_ values of the positive control Vc were 5.538 ± 0.11 μg/mL and 5.369 ± 0.86 μg/mL, respectively. The EA fraction also showed the strongest ferric iron reduction ability, with a FRAP value of 10.655 ± 0.77 mM Fe^2+^/g, which was no significant difference with Vc (14.024 ± 1.30 mM Fe^2+^/g). This deviated slightly from the findings presented by Zhang (Zhang et al., 2021), which might be related to the total flavonoids in *R. aesculifolia* (the *Rodgersia* genus), and exhibited a moderately higher FRAP values compared to Vc. Among the three antioxidant capacity assays, the CE fraction also showed better antioxidant activity, followed by the H_2_O fraction, while the other extracts (DCM and n-Hex) showed moderate antioxidant capacities. Considering that the EA fraction showed the strongest antioxidant capacity compared to other fractions, this result aligned with Yan’s findings (Yan et al., 2017) from the antioxidant activity screening of the *Rodgersia aesculifolia*, thereof the EA fraction was chosen for subsequent studies.

### 3.2 Total phenolic and flavonoid content

Numerous studies have demonstrated that polyphenols and flavonoids were the primary compounds with antioxidant properties, interacting with free radicals before they attack cells, thereby preventing further cellular damage (Lobo et al., 2010; Sha et al., 2021). Results from free radical scavenging experiments showed that the EA fraction had the strongest DPPH and ABTS free radical scavenging abilities, indicating that *R. podophylla* exhibited potential antioxidant activity. To further explore the potential compound types in *R. podophylla*, the TPC and TFC contents of five samples were determined using the Folin-Ciocalteu method and the aluminum nitrate colorimetric method. As shown in Table 1, the EA fraction displayed the highest TPC of 70.984 ± 3.49 mg GAE/g, followed by the CE fraction (41.970 ± 4.14 mg GAE/g). The lowest TPC was n-Hex fraction at 13.094 ± 0.06 mg GAE/g.

**TABLE 1:** Total phenolic and total flavonoid contents of *R. podophylla*.

Extracts	TPC(mg GAE/g dw)	TFC(mg RE/g dw)
CE	41.970 ± 4.14 ^b^	595.326 ± 4.14 ^b^
n-Hex	13.094 ± 0.06 ^c^	99.660 ± 1.96 ^c^
DCM	28.915 ± 1.15 ^b^	87.472 ± 0.51 ^d^
EA	70.984 ± 3.49 ^a^	1,026.096 ± 8.11 ^a^
H_2_O	23.442 ± 0.53 ^b^	77.650 ± 0.13 ^d^

Note: The data were expressed as means ± SD, Means labeled by different letters (a-d) were significantly different at a level of *p <* 0.05 (n = 3) by DMRT (Duncan’s multiple range test).

In addition, the TFC in the EA fraction (1,026.096 ± 8.11 mg RE/g) was also the highest among the five samples, followed by the CE fraction (595.326 ± 4.14 mg RE/g). The lowest TFC was 77.65 ± 0.13 mg RE/g at the H_2_O fraction, which was about 1/13 of the EA fraction. These results further validated our previous findings and provided clues for exploring potential bioactive components in EA fraction with significant antioxidant activity.

### 3.3 Correlation analysis between antioxidant activities and phytochemical components

A comprehensive correlation analysis was executed to thoroughly assess the correlation between the antioxidant activity (DPPH, ABTS, FRAP) of *R. podophylla* and its TPC and TFC. The results are presented in Table 2. On the one hand, substantial correlations were observed among the three antioxidant activity determination. The Pearson correlation coefficients (*R* ^2^) between DPPH with ABTS and FRAP were notably high at 0.998 (*p* < 0.01) and −0.838 (*p* < 0.05), respectively. Furthermore, the *R* ^2^ value between ABTS and FRAP was −0.812 (*p* < 0.05). These findings suggested that these three methodologies were not only reliable but also interchangeable. On the other hand, there were also strong correlations between antioxidant activities and chemical compositions. The FRAP values showed an closely positive correlation with the TPC and TFC at 0.960 (*p* < 0.01) and 0.991 (*p* < 0.01), respectively. While the DPPH and ABTS values were highly negative correlated with TPC (the *R* ^2^ value at −0.795 and −0.772) and TFC (the *R* ^2^ value at −0.783 and −0.749), respectively. The *R* ^2^ value between theTPC and TFC was found to be 0.956 (*p* < 0.01), indicating an exceptionally robust correlation. Synthesizing these findings, it was discerned that polyphenols and flavonoids could potentially be the primary contributors to the antioxidant activity exhibited by *R. podophylla*.

**TABLE 2:** Pearson correlation coefficients (*R* ^2^) among the antioxidant activities and phytochemical contents of *R. podophylla*.

	ABTS	FRAP	TPC	TFC
DPPH	0.998^**^	−0.838^*^	−0.795	−0.783
ABTS		−0.812^*^	−0.772	−0.749
FRAP			0.960^**^	0.991^**^
TPC				0.956^**^

Note: *and ** indicate the correlation is significant at the level of *p* < 0.05 and *p* < 0.01, respectively. DPPH, 2,2-diphenyl-1-picrylhydrazyl; ABTS, 2,2′-azino-bis(3-ethylbenzothiazoline-6-sulphonic acid); FRAP, ferric-reducing antioxidant power; TPC, total phenolic content; TFC, total flavonoid content.

### 3.4 Screening for SOD and XOD ligands in *R. podophylla* with UF-LC-MS

**FIGURE 2:**
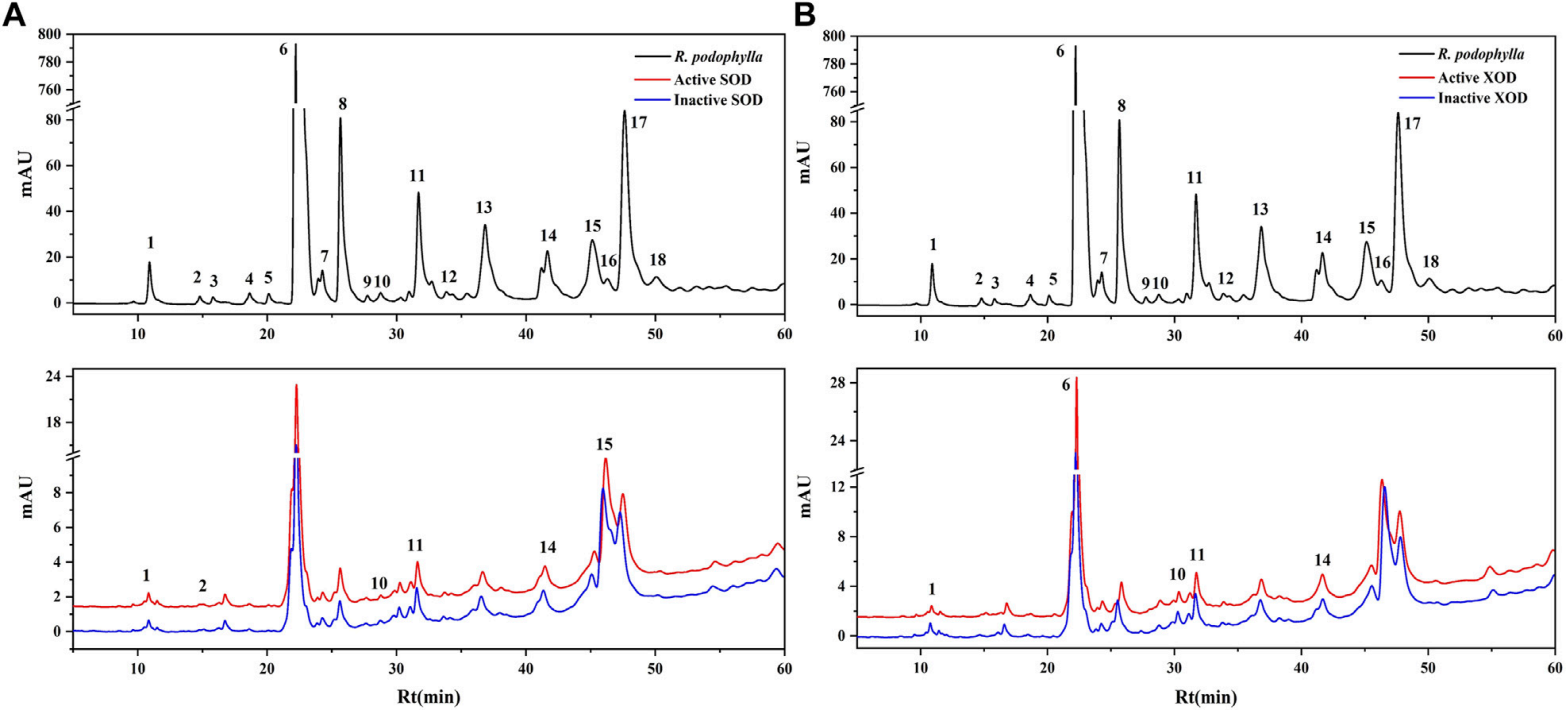
HPLC chromatograms of the chemical constituents in the EA fraction of *Rodgersia podophylla* were obtained after ultrafiltration at 310 nm. The black solid line represents HPLC profiles of the EA fraction of *R. podophylla* without ultrafiltration; the red line and blue line represent the total extract of *R. podophylla* with activated and inactivated SOD **(A)**, XOD **(B)**, respectively.

Investigations have been conducted to explore the antioxidant and anti-hyperuricemic properties of *R. podophylla* through its various extracts (Wang et al., 2012). To further investigate the bioactive constituents that contribute to the antioxidant and anti-hyperuricemic effects in the EA fraction, SOD and XOD were thus selected as the target enzymes for expeditious screening via bioaffinity ultrafiltration. The ligands of SOD and XOD ascertained through this methodology, might potentially constitute active compounds with antioxidant and anti-hyperuricemic properties. As depicted in Figures 2A, B, the results of the ultrafiltrate analysis via HPLC revealed 6 and 5 peaks, respectively, exhibiting varying degrees of binding capacities to SOD and XOD. In this study, the enrichment factor (EF) was employed to express the affinity between the enzyme and the ligand, calculated using the following formula:

$$EF\;\left(\%\right)=\frac{A_{1}\;–\;A_{2}}{A_{0}}\times 100\%$$

Where A_1_, A_2_, and A_0_ represent the peak areas of each chromatographic peak from the EA fraction of *R. podophylla* treated with activated, inactivated, and without SOD or XOD, respectively (Yan et al., 2017; Chen et al., 2020c; Feng et al., 2021). If the peak area of the active group exceeds that of the inactive group, it can be inferred that the group may be classified as a potential inhibitor of the target enzyme (Jiao et al., 2019). When utilized to assess the affinity between the active constituents and the enzyme, the EF value denoted the capacity of various components to bind to the target enzyme.

The EF values of potential SOD and XOD ligands in the EA fraction were summarized in Table 3. For SOD, peak 2 displayed the highest EF value (3.35%), followed by peak 11 (2.33%), peak 10 (1.28%), peak 15 (1.17%), peak 14 (0.96%), and peak 1 (0.54%). For XOD, peak 1 exhibited the highest binding force to XOD, with an EF value of 1.00%, followed by peak 14 (0.55%), peak 11 (0.49%), peak 6 (0.33%), and peak 10 (0.31%). It was observed that 6 and 5 components in the HPLC chromatograms incubated with active SOD and XOD in *R. podophylla* exhibited higher peak areas than those of the inactivated control group, respectively. The findings suggested that these constituents exhibited specific binding affinities towards SOD or XOD, and were therefore consid-ered as primary potential ligands for these enzymes.

**TABLE 3:** The identification, enrichment factor (EF) and the UF-LC-MS/MS data of potential SOD and XOD ligands screened out from *R. podophylla*.

Peak NO.	Rt^a^ (min)	[M-H]^-^	Characteristic fragment (m/z)	Identification	EFs^b^ (%)	
					SOD	XOD
1	10.90	169.26	169, 125, 97	Gallic acid^c^	0.54	1.00
2	14.78	313.46	313, 193, 189	Norbergenin^d^	3.35	-
6	22.20	327.20	327.30, 234, 193, 192, 207	Bergenin^c^	-	0.33
10	28.76	577.35	407, 289, 125	Procyanidin B2^c^	1.28	0.31
11	31.69	289.18	245, 203,151	Catechin^c^	2.33	0.49
14	41.65	479.38	326, 312, 207, 193, 169,125	11-O-Galloylbergenin^d^	0.96	0.55
15	45.12	479.42	327, 313, 207, 193, 169	4-O-Galloylbergenin^d^	1.17	-

^a^ Rt, retention time.

^b^ EFs, enrichment factors.

^c^ Compared with the corresponding standards.

^d^ Identified based on the published literature.

### 3.5 Identification of SOD and XOD ligands in *R. podophylla* with HPLC-UV/ESI-MS/MS

**FIGURE 3:**
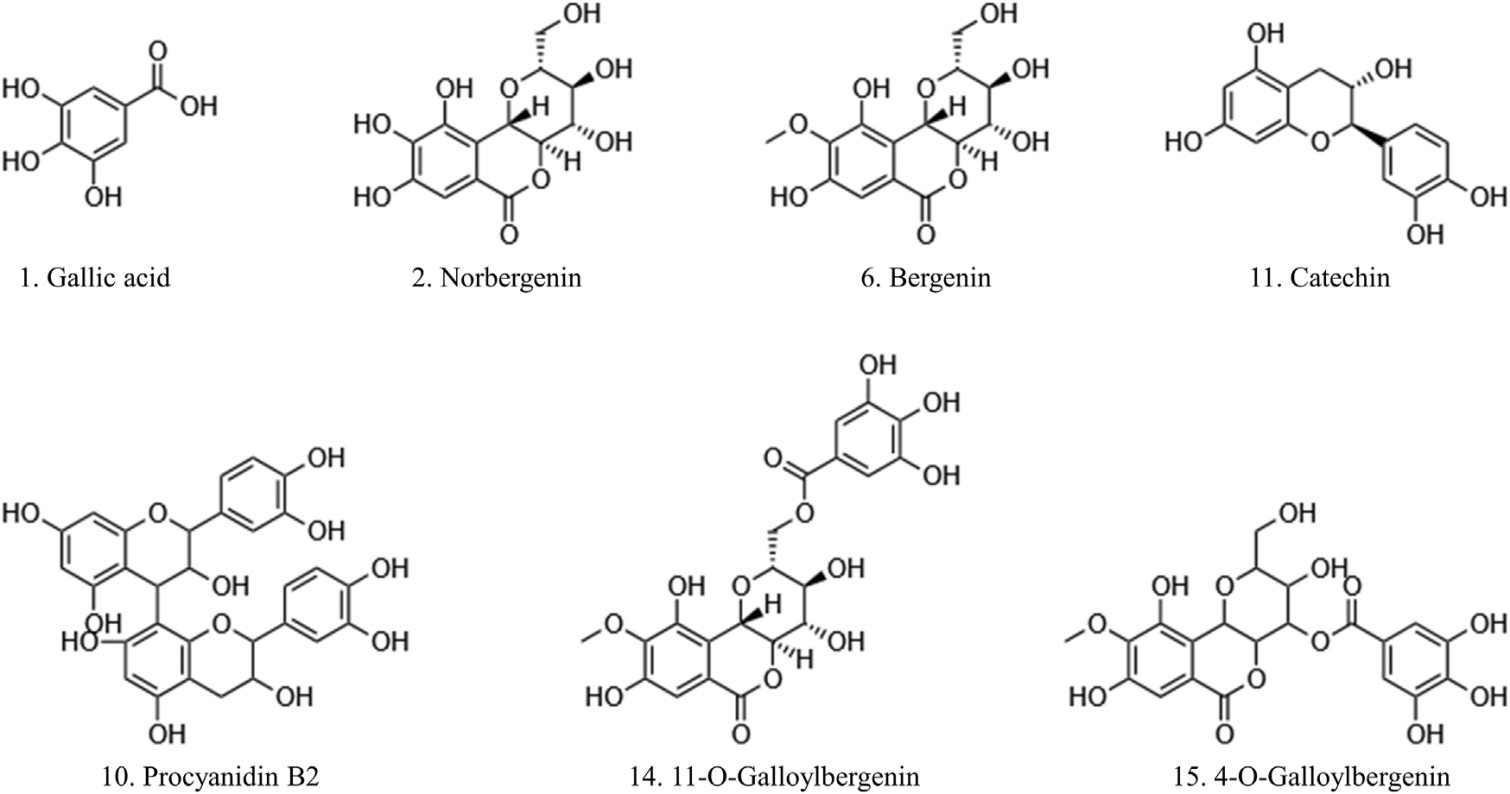
The potential ligands screened from the EA fraction of *R. podophylla* by UF-LC-MS with superoxide dismutase (SOD) and xanthine oxidase (XOD).

The predominant peaks of the EA fraction were identified and characterized using HPLC-UV/ESI-MS/MS. The MS and MS/MS data for these peaks, including retention time (RT), deprotonated molecular ion [M-H]^-^, and representative MS/MS spectral fragments, were presented in Table 3. Furthermore, the structures of the compounds in the EA fraction of *R. podophylla* were tentatively identified, and some representative components were depicted in Figure 3.

Peak 1 exhibited a deprotonated molecular ion at m/z 169.26 [M-H]^-^, with characteristic fragment ions at m/z 125 and 97. Based on its exact mass, fragment ions, and retention time, as well as comparison with the standard, it was identified as gallic acid (Ren et al., 2021). Previous studies also demonstrated that this compound possessed significant antioxidant activity (Badhani et al., 2015; Yan et al., 2017), suggesting that Peak 1 might contribute partly to the antioxidant activity of *R. podophylla*.

Peak 6 displayed a deprotonated molecular ion [M-H]^-^ at m/z 327.20, with a formula of C_14_H_15_O_9_. Four major fragment ions of m/z 234 [M-H-C_2_H_6_O_3_-CH_3_]^-^, m/z 207 [M-H-C_4_H_8_O_4_]^-^, m/z 193 [M-H-C_5_H_10_O_4_]^-^, and m/z 192 [M-H-CH_3_-C_4_H_8_O_4_]^-^ were observed in the MS/MS spectrum. By comparison with previous literature and standard, Peak 6 was identified as bergenin (Li et al., 2013).

Peak 2 exhibited a deprotonated molecular ion [M-H]^-^ at m/z 313.46. A major fragment ion at m/z 193 [M-H-C_4_H_8_O_4_]^-^ was consistent with the fragmentation mechanism of bergenin. Thus, its deprotonated molecular ion [M-H]^-^ at m/z 313 represented bergenin with the loss of a methyl. Based on this, Peak 2 was identified as norbergenin (Ren et al., 2021).

Peak 10 produced a deprotonated molecular ion at m/z 577.35, with a formula of C_30_H_25_O_12_. Its characteristic fragment at m/z 407 corresponded to the fragment ion at m/z 425 [C_22_H_17_O_9_]^-^, which was generated by the cleavage of procyanidins via a Retro-Diels-Alder reaction (RDA), followed by the removal of a portion of water to form the fragment at m/z 407 [C_22_H_17_O_9_-H_2_O]^-^. Another characteristic fragment, at m/z 289, corresponded to the cleavage of m/z 577 [M-H]^-^ into the catechin characteristic fragment at m/z 289 [C_15_H_13_O_6_]^-^. Additionally, m/z 577 also underwent C-ring cleavage to produce the characteristic fragment at m/z 125 [C_6_H_5_O_3_]^-^, which was also present in the fragment ion of peak 10. Through comparison with standards, peak 10 was ultimately determined as procyanidin B2 (Xiao et al., 2017).

Peak 11 presented a deprotonated molecular ion at m/z 289.18 [M-H]^-^. A prominent fragment at m/z 245 [M-H-CO_2_]^-^ resulted from the loss of the CO_2_ group (44 Da) from m/z 289, which then further lost -C_2_H_2_O to generate m/z 203 [M-H-CO_2_-C_2_H_2_O]^-^. Namely, it could be inferred that peak 11 represented either catechin or epicatechin. Additionally, the fragment ion at m/z 151 [M-H-C_6_H_6_O_2_-CO]^-^ corresponded to the characteristic fragment ions generated by the cleavage of catechin or epicatechin via C-ring RDA in negative ion mode. Through comparison with standards, peak 11 was ultimately determined to be catechin (Liu et al., 2009; Ren et al., 2021). Previously, catechin isolated from *R*. *aesculifolia* exhibited its noteworthy DPPH free radical scavenging ability with an IC_50_ of 3.8231 μg/mL, showing stronger antioxidant activity than Vc (IC_50_ = 7.1391 μg/mL) (Yan et al., 2017).

Both peaks 14 and 15 exhibited the same deprotonated molecular ion at m/z 479.42 [M-H]^-^, indicating that they were isomers with the formula C_21_H_20_O_13_. The fragment ion at m/z 327 was characteristic of bergenins, while the fragment ions at m/z 169 [C_7_H_5_O_5_]^-^ and m/z 125 [C_6_H_5_O_3_]^-^ were characteristic of gallic acid. Thus, it can be inferred that peaks 14 and 15 represented monogalloyl bergenin. The fragment ion at m/z 327 represented [M-H-galloyl]^-^, while the fragment ion at m/z 312 represented [M-H-galloyl-CH_3_]^-^, m/z 207 represented [M-H-galloyl-C_4_H_8_O_4_]^-^, and m/z 193 represented [M-H-galloyl-C_5_H_10_O_4_]^-^. In light of the available literature, it can be speculated that the phytochemical composition of genus *Rodgersia* may be galloylbergenin at the 3, 4 or 11 position. Based on the different retention times, peak 14 and peak 15 were inferred to be 11-O-galloylbergenin and 4-O-galloylbergenin, respectively (Ren et al., 2021).

### 3.6 Molecular docking

**FIGURE 4:**
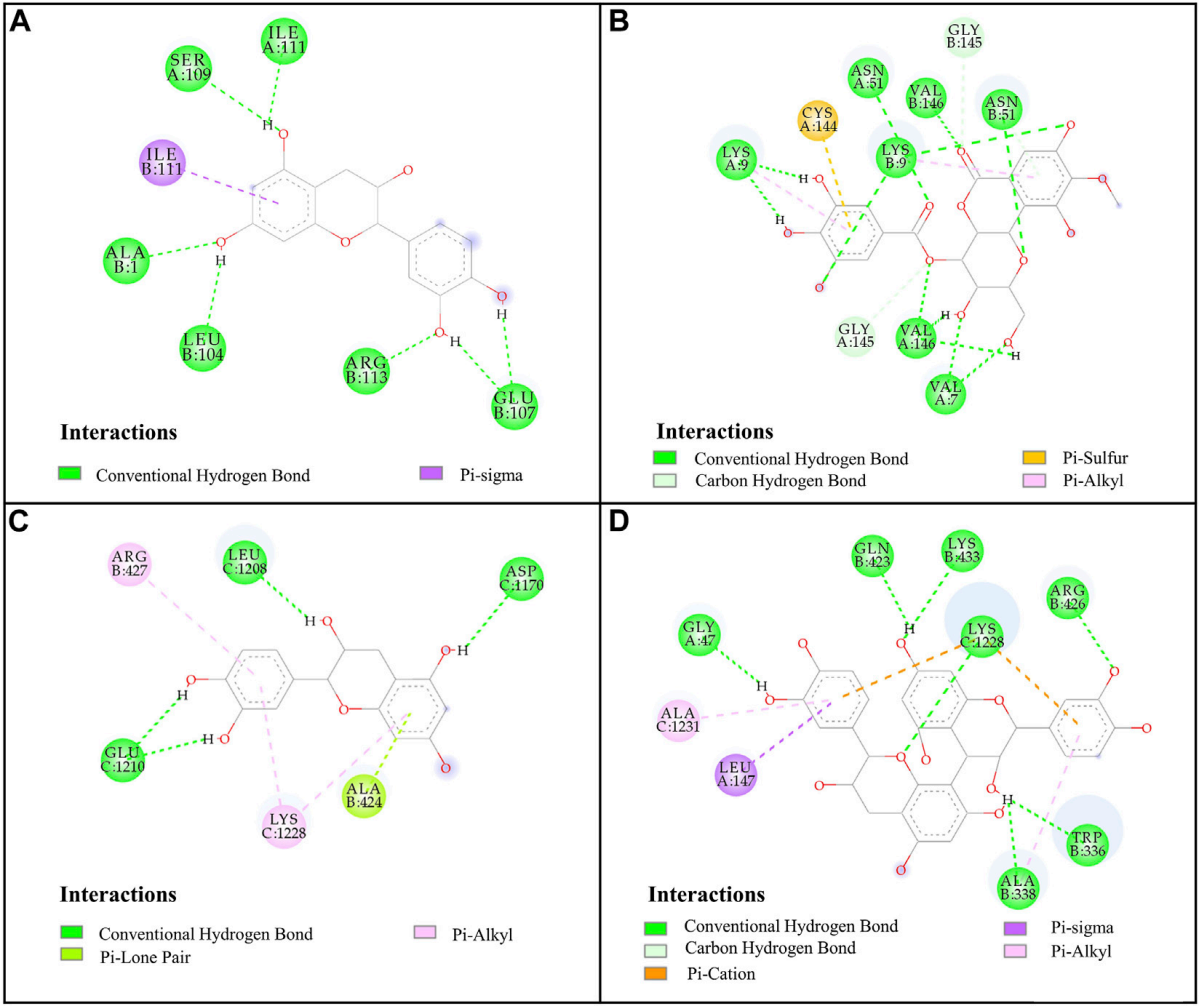
Docked complexes of SOD and XOD: **(A)**, SOD-catechin; **(B)**, SOD-4-O-galloylbergenin; **(C)**, XOD-catechin; **(D)**, XOD-procyanidin B2.

Molecular docking is a widely used technique for evaluating the interactions between enzymes and potential ligands, which could reveal possible interaction patterns by identifying their docking energies, sites of action, and key residues of the receptor (Chen et al., 2020b). Based on the binding degree in affinity ultrafiltration, peaks 2, 11, 10, 15, 14, and 1 were selected as SOD ligands, while peaks 1, 14, 11, 6, and 10 were selected as XOD ligands for molecular docking, respectively. Table 4 summarized the binding energy (BE), inhibition constant (Ki), and amino acids involved in hydrogen bonding. The optimal docking conformation within the binding site was illustrated in Figure 4. Di-thiocarbamate (DTC) and allopurinol (ALL) were used as positive controls for SOD and XOD, respectively.

**TABLE 4:** The molecular docking results of the potential ligands in *Rodgersia podophylla* with SOD and XOD.

Peak	SOD (PDB 1CBJ)			XOD (PDB 1FIQ)		
	BE (kcal/mol)	Ki(μM)	Hydrogen bonds	BE (kcal/mol)	Ki(μM)	Hydrogen bonds
1	−5.06	196.50	Val146, Val7, Lys9	−4.9	255.89	Gln423, Lys433, Lys1128
2	−5.71	65.32	Val146, Val7, Ile149, Asn51	ND	ND	ND
6	ND	ND	ND	−5.26	139.25	Ile1229, Ser1234, Gly47
10	−4.67	379.44	Val146, Val7, Asn51, Gly148	−8.08	1.20	Arg426, Lys433, Gln423, Gly47
11	−6.85	9.51	Ala1, Ile111, Leu104, Glu107	−8.54	0.55	Glu1210, Leu1208, Asp1170
14	−5.99	40.42	Val146, Val7, Lys9, Cys144	−5.93	45.18	Gln423, Lys433, Arg426, Ala338
15	−6.67	13.01	Val146, Val7, Asn51, Lys9	ND	ND	ND
DTC^a^	−4.02	1,130.00	Val146, Gly49	ND	ND	ND
ALL^b^	ND	ND	ND	−5.03	206.23	Arg426, Glu1210

PDB, protein data bank; 1CBJ, the crystal structure accession number of SOD; 1FIQ, the crystal structure accession number of XOD; BE, binding energy; Ki, inhibition constant; a, Dithiocarbamate (DTC), positive control of SOD; b, Allopurinol (ALL), positive control of XOD; ND, not detected; Val, valine; Lys, lysine; Ile, isoleucine; Asn, asparaginate; Gly, glycine; Ala, alanine; Leu, leucine; Glu, glutamic acid; Cys, cysteine; Gln, glutamine; Ser, serine; Arg, arginine; Asp, aspartic acid.

For SOD, the six compounds screened by bioaffinity ultrafiltration were lower than that of the positive control DTC (BE, −4.02 kcal/mol; Ki, 1,130.00 μM), which indicated that these compounds exhibited strong interactions with SOD. Among them, peak 11 (catechin, BE, −6.85 kcal/mol; Ki, 9.51 μM) displayed the strongest affinity with SOD, followed by peak 15 (4-O-galloylbergenin, BE, −6.67 kcal/mol; Ki, 13.01 μM), peak 14 (11-O-galloylbergenin, BE, −5.99 kcal/mol; Ki, 40.42 μM), peak 2 (norbergenin, BE, −5.71 kcal/mol; Ki, 65.32 μM), peak 1 (gallic acid, BE, −5.06 kcal/mol; Ki, 196.50 μM), and peak 10 (procyanidin B2, BE, −4.67 kcal/mol; Ki, 379.44 μM). These results were generally consistent with those obtained from bioaffinity ultrafiltration (Table 4). Through the visualization of molecular docking results, it was found that there was a pi-sigma and numerous hydrogen bonds between peak 11 (catechin) and SOD. As shown in Figure 4A, Figure 7 hydrogen bonds were formed between catechin and amino acid residues such as Ala1, Ile111, Leu104 and Glu107. As depicted in Figure 4B, peak 15 (4-O-galloylbergenin) formed 12 hydrogen bonds and engaged in additional interactions, such as pi-sulfur and pi-alkyl, with amino acid residues including Val146, Val7, Asn51, and Lys9. Consequently, the stability of protein-ligand complexes was not only attributed to hydrogen bonding but also to other interaction forces.

For XOD, peak 11 (catechin) exhibited the highest interaction with XOD, with the lowest BE value of −8.54 kcal/mol and the Ki value of 0.55 μM. Figure 4C showed that catechin formed 4 hydrogen bonds with amino acid residues such as Glu1210, Leu1208 and Asp1170 of XOD and formed pi-alkyl with residues. Peak 10 (procyanidin B2, BE, −8.08 kcal/mol; Ki, 1.2 μM, Figure 4D) formed hydrogen bonds with XOD through amino acid residues such as Arg426, Lys433, Gln423, and Gly47. The presence of pi-cation, pisigma and pi-alkyl interactions increased its affinity with XOD. In addition, peak 14 (11-O-galloylbergenin, BE, −5.93 kcal/mol; Ki, 45.18 μM) and peak 6 (bergenin, BE, −5.26 kcal/mol; Ki, 139.25 μM) also exerted higher activity than the positive control ALL (BE, −5.03 kcal/mol; Ki, 206.23 μM), indicating that these compounds also had good interactions with XOD.

In summary, peaks 11, 15, 14, 2, 1, and 10 for SOD and peaks 11, 10, 14, 6 and 1 for XOD were identified as potential ligands that warranted further investigation. Their structures were shown in Figure 3, and their retention times and mass spectral fragments were presented in Table 3.

### 3.7 Anti-hyperuricemic capacity of potential ligands by XOD inhibition experiments

**FIGURE 5:**
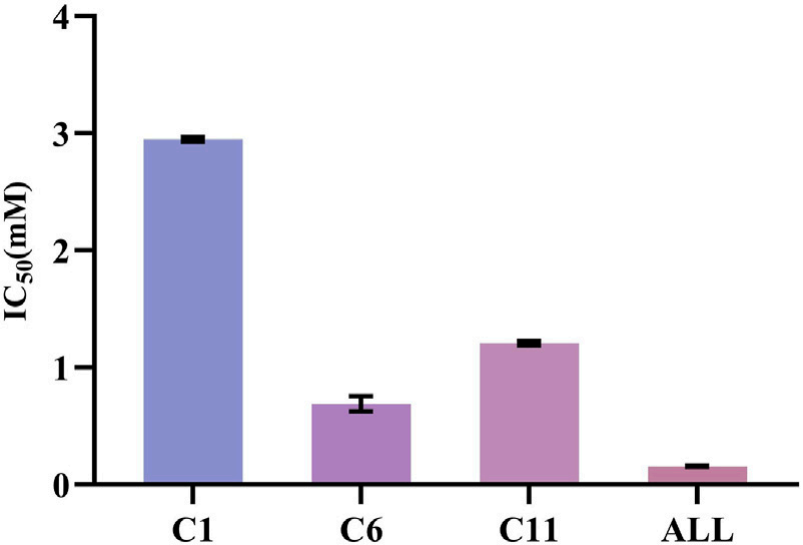
The IC_50_ of potential ligands with XOD. C1: gallic acid; C6: bergenin; C11: catechin; ALL: allopurinol.

The XOD inhibition assay was conducted with a slightly modified methodology previously described by Wang et al. (Wang et al., 2012). At the concentration of 0.25 mg/mL, the *R*. *aesculifolia* extract achieved an XOD inhibition rate of 72.79%. This rate increased to an impressive 85.18% when the concentration was elevated to 1.25 mg/mL. Meanwhile, the XOD inhibitory activity *in vitro* of the compounds, identified through UF-LC-MS screening, was further confirmed. As shown in Figure 5, compound 6 (bergenin) displayed the strongest inhibitory effect on XOD, with an IC_50_ value of 0.680 ± 0.061 mM, which was comparable with the positive control drug, allopurinol (IC_50_ = 0.156 ± 0.005 mM) (*p* > 0.05). Compound 11 (catechin) and compound 1 (gallic acid) also showed attractive inhibitory effects, with IC_50_ values of 1.208 ± 0.015 mM and 2.949 ± 0.016 mM, respectively. To this end, the present results not only confirmed the significant XOD inhibitory activity of the compounds screened through bio-affinity ultrafiltration, but also provided a foundation for further investigation into the anti-hyperuricemia properties of *R. podophylla*.

## 4 Conclusion

In this study, the antioxidant capacities of *R. podophylla* extracts were firstly assessed using DPPH, ABTS, and FRAP assays. The EA fraction of *R. podophylla* exhibited the highest radical scavenging and ferric reduction abilities, with IC_50_ values of 3.863 ± 0.34 μg/mL and 5.537 ± 0.38 μg/mL for DPPH and ABTS, and the FRAP value of 10.655 ± 0.77 mM Fe^2+^/g, respectively. Additionally, the EA fraction exhibited the highest total phenolic and flavonoid contents among all fractions, with values of 70.984 ± 3.49 mg GAE/g dw and 1,026.096 ± 8.11 mg RE/g dw, respectively. A bio-affinity ultrafiltration coupled with LC-MS/MS strategy with SOD and XOD as target enzymes was employed to screen potential antioxidant and anti-hyperuricemic compounds from the EA fraction. Based on the binding capacities, norbergenin (peak 2), catechin (peak 11), procyanidin B2 (peak 10), 4-O-galloylbergenin (peak 15), 11-O-galloylbergenin (peak 14), and gallic acid (peak 1) were identified as potential SOD ligands, while gallic acid (peak 1), 11-O-galloylbergenin (peak 14), catechin (peak 11), bergenin (peak 6), and procyanidin B2 (peak 10) were identified as potential XOD ligands, respectively. Furthermore, molecular docking revealed that these seven compounds exhibited favorable interactions with SOD and XOD through hydrogen bonding and other types of intermolecular forces. Notably, their binding affinities even surpassed those of the positive controls (DTC and ALL). Thereafter, the XOD inhibition assays *in vitro* discerned that all these molecular entities demonstrated strong XOD inhibition, especially bergenin and catechin. In summary, this study represented the first application of the UF-LC-MS method for the rapid screening of bioactive compounds in *R. podophylla* extracts, specifically targeting their antioxidant and anti-hyperuricemia properties. The research not only provided novel evidence to support the antioxidant and anti-hyperuricemic properties of *R. podophylla*, but also clarified the potential bioactive ligands for its further research and application. These bioactive compounds hold promise for future development as health supplements or natural medicines for the prevention and treatment of oxidative stress and hyperuricemia-associated diseases.

## Data Availability

The original contributions presented in the study are included in the article/Supplementary material, further inquiries can be directed to the corresponding authors.
